# Mechanical failure of articulating polymethylmethacrylate (PMMA) spacers in two-stage revision hip arthroplasty: the risk factors and the impact on interim function

**DOI:** 10.1186/s12891-019-2759-x

**Published:** 2019-08-14

**Authors:** Fu-Shine Yang, Yu-Der Lu, Cheng-Ta Wu, Kier Blevins, Mel S. Lee, Feng-Chih Kuo

**Affiliations:** 1 Department of Orthopedic Surgery, Kaohsiung Chang Gung Memorial Hospital, No 123, Ta Pei Road, Niao Sung Dist, Kaohsiung, 833 Taiwan; 2 Department of Orthopaedic Surgery, Duke University Medical Center, Box 3000, Durham, NC 27710 USA

**Keywords:** Hip, PMMA, Articulating spacer, Complications, Periprosthetic joint infection

## Abstract

**Background:**

This study aimed to investigate the risk factors for mechanical failure of cement spacers and the impact on hip function after two-stage exchange arthroplasty for periprosthetic joint infection (PJI).

**Methods:**

Thirty-one patients (19 males and 12 females) with hip PJIs underwent resection arthroplasty and implantation of cement spacers from January 2014 to December 2015. Patients who encountered spacer-associated mechanical complications in the interim period (14 of 31) were compared with those without complications (17 of 31). Complications were defined as spacer dislocation, spacer fracture, spacer fracture with dislocation, and femoral fracture during or following spacer implantation. Hip functional outcome was assessed using the Harris hip score (HHS). Treatment success was defined according to the following criteria: (1) no symptoms or signs indicative of infection; (2) no PJI-related mortality; and (3) no subsequent surgical intervention for infection after reimplantation surgery. Multivariate logistic regression and Kaplan-Meier survival curves were used for analysis.

**Results:**

Fourteen patients (14/31 = 45%) suffered at least one spacer-related complication within the interim period. The development of spacer complications was associated with a younger age (odds ratio [OR] 0.91, 95% confidence interval [CI] 0.83–1.00, *p* = 0.045) and chronic PJI (OR 14.7, 95% CI 1.19–182, *p* = 0.036). Patients with spacer complications also had a lower median HHS (37 vs. 60, *p* < 0.001) before reimplantation in comparison to those without spacer complications. After reimplantation, the two groups had a similar median HHS (90 vs. 89, *p* = 0.945). Two patients did not undergo reimplantation due to extensive comorbidities, and subsequently retained the antibiotic spacer for definitive treatment. The 2-year treatment success rate was 84.6% in the spacer-complication group and 87.5% in the non-spacer-complication group (*p* = 0.81).

**Conclusion:**

There was a high complication rate for articulating PMMA spacers during the interim period of two-stage revision total hip arthroplasty. A young age and chronic infection were the primary risk factors associated with mechanical complications. Patients at high risk of spacer-related mechanical complications should be advised accordingly by surgeons. Knowing the possible risk factors, surgeons should educate patients thoroughly to avoid spacer complications, thereby increasing patient satisfaction in the interim stage.

**Level of evidence:**

Prognostic Level III.

## Background

Periprosthetic joint infection (PJI) is a rare but devastating complication of total hip arthroplasty (THA). The gold standard for treating PJI after THA is via two-stage revision arthroplasty, which involves initial removal of the prosthesis and insertion of an articulating polymethylmethacrylate (PMMA) spacer. The second stage of reimplantation consists of insertion of a new prosthesis once the underlying infection has been eliminated [1, 2]. There are several kinds of antibiotic-loaded cement used between stages that have led to favorable success rates, ranging from 82 to 96% [3–8].

Although antibiotic cement beads provide a high local concentration of antibiotics [9], they present numerous disadvantages, including joint instability, increased energy demand during gait, soft-tissue contractures and abnormal stress on the spine from pseudoarthrosis [3, 9]. Articulating PMMA spacers have been shown to eliminate infection at a rate similar to cement beads [4]; however, articulating spacers are advantageous in comparison with beads, in that they provide temporary soft tissue tension and improved hip scores, and allow for increased functional capacity [2]. Additionally, application of an articulating spacer is relatively straight-forward during reimplantation surgery [7], and results in a lower dislocation rate postoperatively in comparison to antibiotic beads [4]. In circumstances in which the patient’s condition does not allow for revision surgery, the articulating PMMA spacer can remain implanted for definitive treatment [10, 11]. In comparison, preformed spacers provide similar infection eradication rates to articulating spacers, as well as good mechanical stability [3, 12]; however, the cost of preformed spacers is much higher than that of articulating PMMA spacers.

At times, mechanical complications may occur following spacer insertion, leading to reduced patient function and mobility. In the first stage, complication rates associated with articulating spacers have been reported to be anywhere from 19.5 % to 50 %[13, 14]. These complications include spacer dislocation, spacer fracture and femoral fracture [15]. To date, it remains unclear whether spacer-associated complications influence the functional outcome following reimplantation. This study aimed to (1) investigate the risk factors for mechanical complications of articulating PMMA spacers, and (2) examine the association between complications arising following implantation of an articulating PMMA spacer and functional outcome following reimplantation. We hypothesized that mechanical complications associated with articulating PMMA spacers might influence the overall functional outcome following reimplantation.

## Methods

### Patient enrollment

**Fig. 1:**
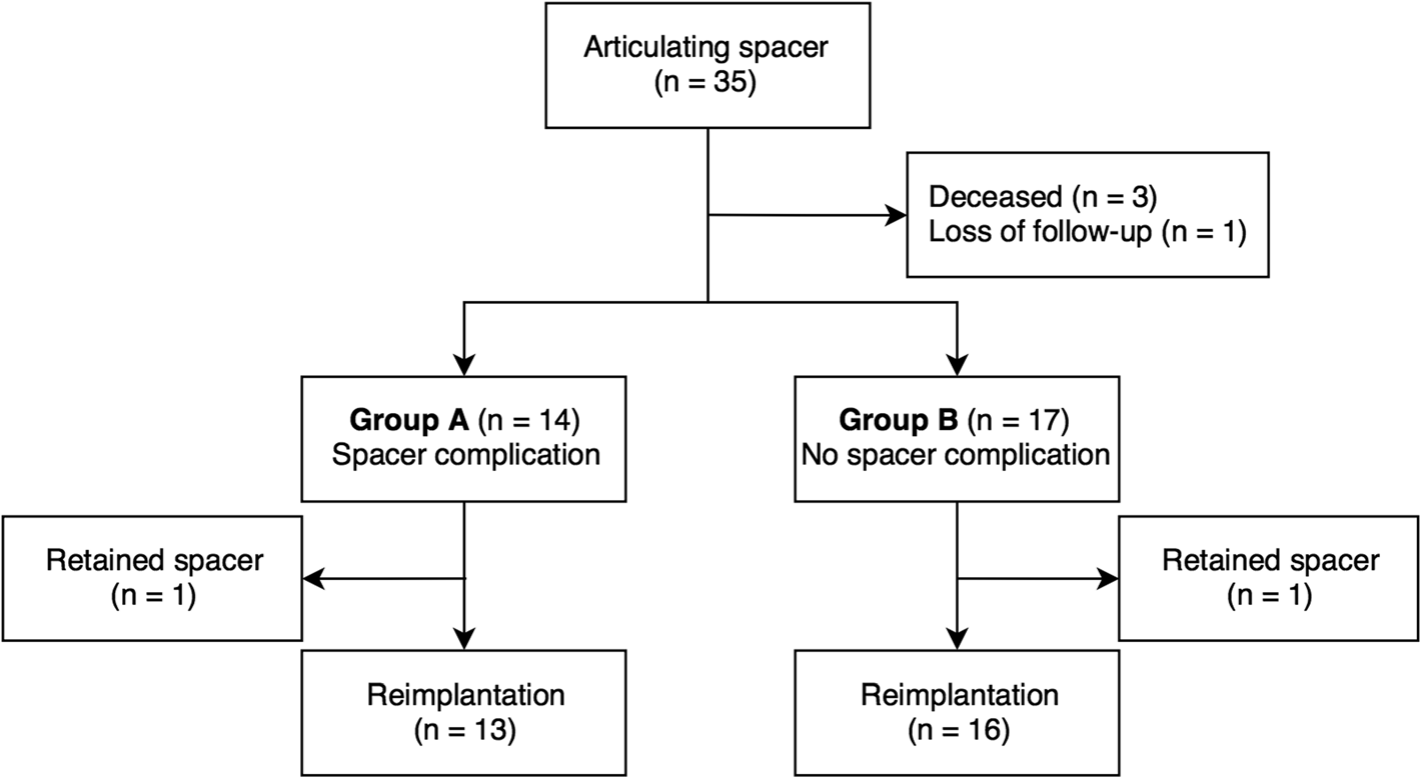
Flowchart of eligible patients

Following Institutional Review Board approval, patients of a single institution who underwent two-stage exchange arthroplasty were enrolled in this study between January 2014 and December 2015. The indications for the two-stage procedure were chronic PJI, acute infection with failed debridement, and implant loosening. Acute infection was defined as occurring within 3 weeks of the procedure, or a late hematogenous infection with development of symptoms within 3 weeks of surgery. Chronic PJI was defined as any infection developing after 3 weeks following surgery [16]. All patients who underwent articulating PMMA spacer implantation were included. The exclusion criteria included any patient who underwent PJI treatment with cement beads, treatment with static spacers, one-stage revision, debridement with or without modular exchange, amputation, or arthrodesis. Patients with megaprosthesis and a history of septic arthritis in the native joint were also excluded. A total of 35 patients who underwent implantation of an articulating PMMA spacer were included in this study initially. One patient was lost to follow-up 4 months after resection arthroplasty, and three patients died from septic shock (unrelated to PJI) within 6 months of resection arthroplasty. Finally, 31 patients (19 men, 12 women) were divided into two groups, those with (group A, *n* = 14) and without spacer-related complications (group B, *n* = 17) (Fig. 1). Patients in both groups were followed-up for a minimum of 2 years after reimplantation.

Medical charts were manually reviewed to obtain information relevant to the clinical course following resection arthroplasty, and the following measures and data were recorded: patient characteristics, American Society of Anesthesiologists (ASA), prosthesis type, comorbidities, classification of PJI (acute or chronic) [16], serologic markers, interval between resection arthroplasty and reimplantation, surgical approach, bone defects, and microorganism cultures.

### Treatment protocol

**Fig. 2:**
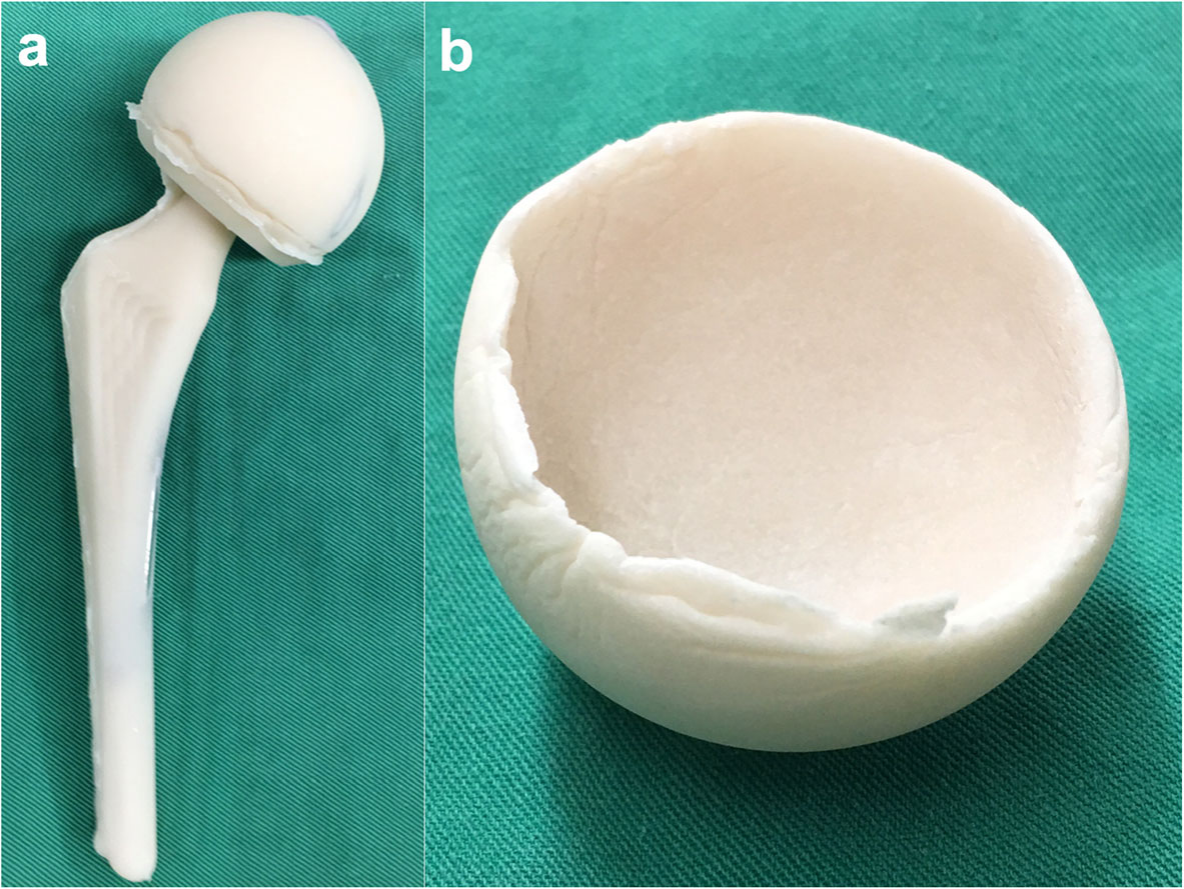
Images showing: **a**) the femoral and **b**) the cup part of a PMMA articulating spacer

The first stage consisted of prosthesis removal and radical debridement, followed by temporary articulating spacer placement. A minimum of three sets of tissue specimens were taken for culture, and custom-made silicon molds were used to model the femoral cement spacer (Fig. 2a). Antibiotic-loaded bone cement was added to the silicon mold with two or three 3-mm Kirschner wires forming the endoskeleton. The cup spacer was shaped using a unipolar cup (Fig. 2b). Local antibiotics in the PMMA bone cement were tailored to the organism previously identified by culture. If the infecting organism was not known at the time of resection arthroplasty, broad coverage with vancomycin and ceftazidime was employed. The antibiotic dose per batch of cement (40 g) was limited to 6–8 g.

The cup part of the articulating PMMA spacer was cemented onto the acetabulum, and the femoral part of the articulating spacer was inserted into the femoral canal, with cement added to the meta-diaphyseal portion of the stem. This enhanced construct fixation to prevent spacer dislocation and decrease the risk of cement incarceration in the femoral canal.

Following the first stage of surgery, a physical therapist was consulted for strengthening on the first postoperative day and protected non-weight-bearing activity on the second postoperative day. Toe-touch weight-bearing was allowed 4–6 weeks after the first stage of surgery. Hip precautions were advised for 6 weeks postoperatively to prevent hip dislocation. Organism-specific antibiotics were administered intravenously for 2 weeks after resection arthroplasty, followed by oral antibiotics for 4 weeks. The serum C-reactive protein (CRP) level and erythrocyte sedimentation rate (ESR) were routinely checked every 2 weeks. Reimplantation was subsequently performed based on the quality of healing of the surgical wound and the following criteria being met: no fistula formation, no drainage, and no elevation of the ESR or CRP level following an antibiotic-free interval of at least 2 weeks. Three sets of deep aerobic and anaerobic bacterial cultures were obtained as routine at the second-stage surgery.

Post-operative empirical antibiotics were prescribed after the second-stage surgery, using the same agent as prior to second-stage surgery, and discontinued after confirmation of a negative culture. Intravenous and oral antibiotics were prescribed for a total of 6 weeks if the culture was positive at the second stage. All the patients undergoing reimplantation were followed-up for a minimum period of 2 years, until death or recurrent infection.

### Outcome measurement

Mechanical complications associated with articulating spacers were defined as spacer dislocation, spacer fracture, spacer fracture with dislocation, and femoral fracture during spacer insertion or during the interim period. Hip function was accessed using the Harris hip score (HHS). Treatment success was defined according to the Delphi consensus criteria proposed by Diaz-Ledezma et al. [17]: (1) infection eradication was characterized by a healed wound without drainage, fistula, or pain, and with no recurrence of infection; (2) no occurrence of PJI-related mortality (e.g., sepsis, necrotizing fasciitis); and (3) no subsequent surgical intervention for infection after reimplantation surgery.

### Statistical analysis

Statistical analysis was performed using MedCalc (version 17.9.2, Ostend, Belgium). Categorical variables were expressed as counts and percentages, and continuous data as medians and interquartile ranges (IQRs). As a preliminary Kolmogorov-Smirnov test did not demonstrate a normal distribution for our small sample size, we decided to use the nonparametric Mann-Whitney U test to compare the continuous variables. Categorical variables were compared using the chi-square test or the Fisher exact test. Multivariate analysis with binary logistic regression was used to determine further risk factors associated with the development of spacer-related complications (*p* < 0.10 in the univariate analysis). A Kaplan-Meier survivorship curve was generated to estimate the proportion of patients in whom treatment was successful in the two groups. The difference in survivorship between the two groups was assessed using the log-rank test. A *p*-value<0.05 was considered statistically significant.

## Results

**Fig. 3:**
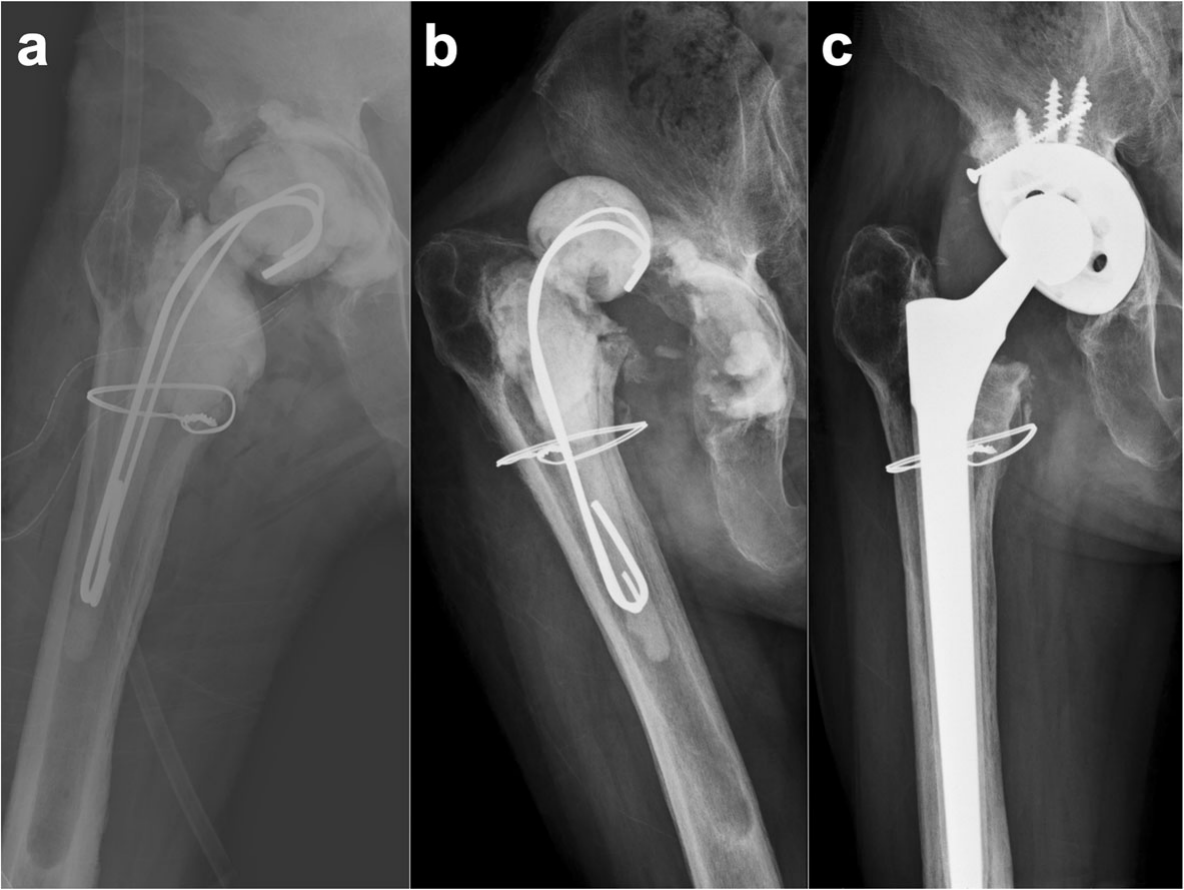
Hip radiographs showing: **a**) post-operative X-ray after implantation of a PMMA articulating spacer; **b**) dislocation of the PMMA articulating spacer 3 months later after implantation; and **c**) a well-fixed cementless total hip arthroplasty 2 years following reimplantation

The median age of the whole cohort of 31 patients was 56 years (interquartile range, IQR: 50–71). The median body mass index (BMI) was 23.7 kg/m^2^ (IQR: 22.1–27.7). The median interval between resection arthroplasty and reimplantation was 14.5 weeks (IQR: 11–23). Preoperative diagnoses before primary arthroplasty included 16 patients with osteoarthritis, 10 patients with femoral neck fractures, and 5 patients with osteonecrosis of the femoral head. Fourteen patients (14/31 = 45%, group A) suffered at least one spacer-related mechanical complication in the interim period. The other patients without mechanical complications (17/31 = 55%) comprised group B (Table 1). Six patients had spacer dislocation (19.4%), of which 4 were treated by closed reduction and immobilization with an abduction brace (Fig. 3); the 2 remaining patients underwent spacer exchange due to failure of closed reduction. Spacer fracture occurred in 3 patients (9.7%), 2 of whom were asymptomatic and were treated with conservative treatment. The remaining patient with a spacer fracture underwent spacer exchange due to recurrent infection. One patient who had a spacer dislocation and fracture underwent spacer exchange (3.2%). Femoral fractures occurred in 4 patients (12.9%); 2 suffered femoral fracture during resection and were treated with plating/wiring fixation, while the remaining 2 with femoral shaft fractures near the spacer stem were treated with conservative treatment.

**Table 1:** Demographic data of patients in the spacer-complication group (group A) and the no-spacer-complication group (group B)

Characteristics	Overall (*n* = 31)		Group A (*n* = 14)		Group B (*n* = 17)		*p*-value
Age, median (IQR), y	56	(50 to 71)	55	(41 to 59)	64	(52 to 77)	0.021
Male, *n* (%)	19	(61.3)	10	(71.4)	9	(52.9)	0.290
BMI, median (IQR), kg/m^2^	23.7	(22.1 to 27.7)	23.5	(21.9 to 29.4)	23.8	(22.6 to 25.7)	0.787
ASA class, *n* (%)							0.436
2	11	(35.5)	6	(42.9)	5	(29.4)	
3	20	(64.5)	8	(57.1)	12	(70.6)	
Prosthesis type, *n* (%)							0.895
Bipolar	8	(25.8)	3	(21.4)	5	(29.4)	
Primary	11	(35.5)	4	(28.6)	7	(41.2)	
Revision	12	(38.7)	7	(50.0)	5	(29.4)	
Comorbidities, *n* (%)							
Diabetes mellitus	9	(29.0)	4	(28.6)	5	(29.4)	0.959
Renal disease	3	(9.7)	2	(14.3)	1	(5.9)	0.431
Liver disease	6	(19.4)	2	(14.3)	4	(23.5)	0.517
Drug abuse	3	(9.7)	2	(14.3)	1	(5.9)	0.431
Alcoholism	3	(9.7)	2	(14.3)	1	(5.9)	0.835
Psychiatric disorder	2	(6.5)	2	(14.3)	0	(0)	0.107
Classification of PJI, n (%)							0.003
Acute	10	(32.3)	1	(7.1)	9	(52.9)	
Chronic	21	(67.7)	13	(92.9)	8	(47.1)	
1st stage							
Pre-op ESR, median (IQR), mm/hr	63	(30 to 91)	62	(28 to 96)	63	(30 to 76)	0.889
Pre-op CRP, median (IQR), mg/dL	15	(6.3 to 73.0)	29	(9.2–95)	14.8	(5.9 to 55.7)	0.578
Approach, n (%)							0.031
Posterior	23	(74.2)	13	(92.9)	10	(58.8)	
Direct lateral	8	(25.8)	1	(7.1)	7	(41.2)	
Paprosky bone defect classification, n (%)							
Acetabulum							0.143
I	14	(45.2)	5	(35.7)	9	(52.9)	
II (A + B + C)	8	(25.8)	6	(42.9)	2	(11.8)	
III (A + B)	9	(29.0)	3	(21.4)	6	(5.3)	
Femur							0.977
I	8	(25.8)	4	(28.6)	4	(23.5)	
II	10	(32.3)	4	(28.6)	6	(35.3)	
III (A + B)	11	(35.5)	5	(35.7)	6	(35.3)	
IV	2	(6.4)	1	(7.1)	1	(5.9)	
Additional spacer exchange	13	(41.9)	6	(42.9)	7	(41.2)	0.953
Recurrent infection	10		3		7		
Interim period^a^, median (IQR), weeks	14.5	(11 to 32)	15	(10 to 20)	13.5	(11 to 27)	0.950
Reimplantation^a^							
Pre-op ESR, median (IQR), mm/hr	29.9	(11.5 to 47.0)	31.2	(14.5 to 46.0)	28.8	(8.3 to 55.5)	0.397
Pre-op CRP, median (IQR), mg/dL	16.7	(1.32 to 5.5)	28.7	(1.83 to 6.1)	6.9	(1.0 to 5.7)	0.156
Follow-up period^a^, median (IQR), months	29	(23 to 35.5)	29	(25 to 34)	30	(23 to 36)	0.948

^a^One patient in group A and one patient in group B had permanent spacer implantation without revision surgery and were excluded from calculation of the interim period, CRP/ESR before reimplantation, and duration of follow-up period

*IQR* interquartile range, *BMI* body mass index, *ASA* American Society of Anesthesiologists, *ESR* erythrocyte sedimentation rate, *CRP* C-reactive protein, *PJI* periprosthetic joint infection

The posterior approach was used in 92.9% of the patients in group A and 58.8% of the patients in group B (*p* = 0.031). Among patients in whom the posterior approach was employed, 7 suffered spacer dislocation. No patient experienced spacer dislocation when the direct lateral approach was used. Among the patients with spacer dislocation, according to the Paprosky classification, bone defects were classified as type I in 3 patients, type IIB in 2 patients, and type IIIA in 2 patients. There was no significant association between Paprosky type and spacer dislocation.

Thirteen patients underwent additional spacer exchange surgery in the interim period, including 6 patients in group A and 7 patients in group B. All 7 patients in group B who did not experience mechanical complications underwent additional spacer exchange surgery due to recurrent infection. Three of the 6 patients in group A had additional surgery for spacer exchange in the interim period owing to recurrent infection, one of whom had simultaneous spacer dislocation. The remaining 3 patients in group A underwent spacer exchange due to recurrent dislocation or fracture of the spacer. The rate of additional spacer exchange for mechanical complications or recurrent infection was not significantly different between the two groups (42.9% vs. 41.2%, *p* = 0.953).

In the whole cohort of 31 patients who were treated with articulating PMMA spacer implantation, gram-positive microorganisms caused the infection in the majority of cases. Coagulase-negative *Staphylococcus* was the most common pathogen (25.8%), followed by *Staphylococcus aureus* (19.4%) and Methicillin-resistant *Staphylococcus aureus* (6.5%). Culture-negative and polymicrobial PJI accounted for 22.6%and 16.1% of cases, respectively (Table 2).

**Table 2:** Microbiologic laboratory data for specimens isolated during resection arthroplasty

Pathogens, *n* (%)		
Coagulase-negative *Staphylococcus*	8	(25.8)
*Staphylococcus aureus*	6	(19.4)
Methicillin-resistant *Staphylococcus aureus*	2	(6.5)
*Streptococcus sanguinis*	1	(3.2)
*Pseudomonas aeruginosa*	1	(3.2)
*Escherichia coli*	1	(3.2)
Polymicrobial	5	(16.1)
Culture-negative	7	(22.6)

Patients who had mechanical complications were younger than those without mechanical complications (*p*=0.021). Chronic infection (*p*=0.003) and utilization of the posterior approach (*p* = 0.031) were risk factors for the development of spacer-related complications according to univariate analysis (Table 1). After adjusting for potential confounders (*p* < 0.10 in the univariate analysis), according to multivariate analysis, only a younger age (OR 0.91, 95% CI 0.83–1.00, *p* = 0.045) and chronic PJI (OR 14.7, 95% CI 1.19–182, *p* = 0.036) remained as risk factors associated with the development of spacer-related complications (Table 3).

**Table 3:** Stepwise binary logistic regression between patients with spacer complications and those without

	Odds ratio	95% CI	*p*-value
Age (y)	0.91	0.83–1.00	0.045
Chronic infection	14.7	1.19–182	0.036

*CI* confidence interval

**Fig. 4:**
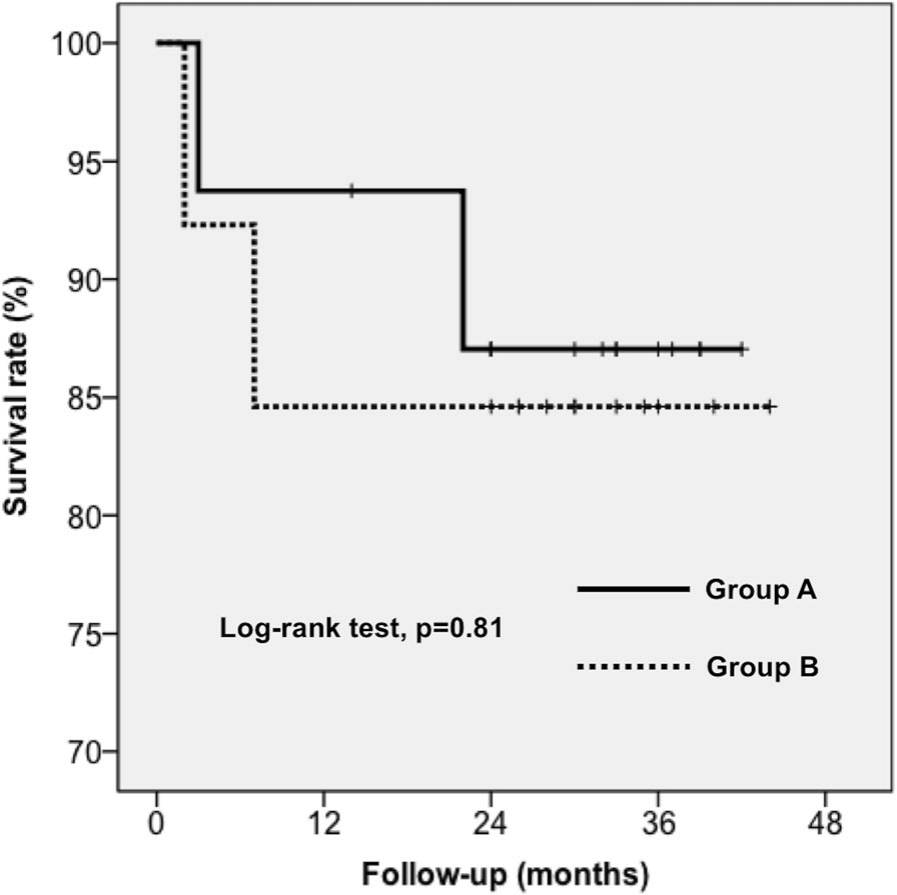
Kaplan-Meier survival curve analysis of patients in whom treatment was successful in the spacer-related-complication group versus the no-spacer-complication group

One patient in each group retained the spacer without further reimplantation (Fig. 1). Twenty-nine patients eventually underwent reimplantation, and only one patient had a positive culture finding following reimplantation, revealing infection with *Staphylococcus lugdunensis*. That patient received intravenous and oral antibiotics for a total of 6 weeks, and no recurrent infection was noted during the follow-up period. One of the 29 patients died 3 months after reimplantation from a cause unrelated to PJI. Among the three patients with recurrent infection after reimplantation, two underwent resection arthroplasty, and the other underwent debridement with prosthesis retention. When stratified by spacer-related complications, the proportion of patients in whom treatment was successful after reimplantation was 84.6% (11/13) in the spacer-related-complications group (group A) versus 87.5% (14/16) in the non-spacer-complications group (group B). There was no statistically-significant difference between the two groups (log-rank test, *p* = 0.81) (Fig. 4).

**Fig. 5:**
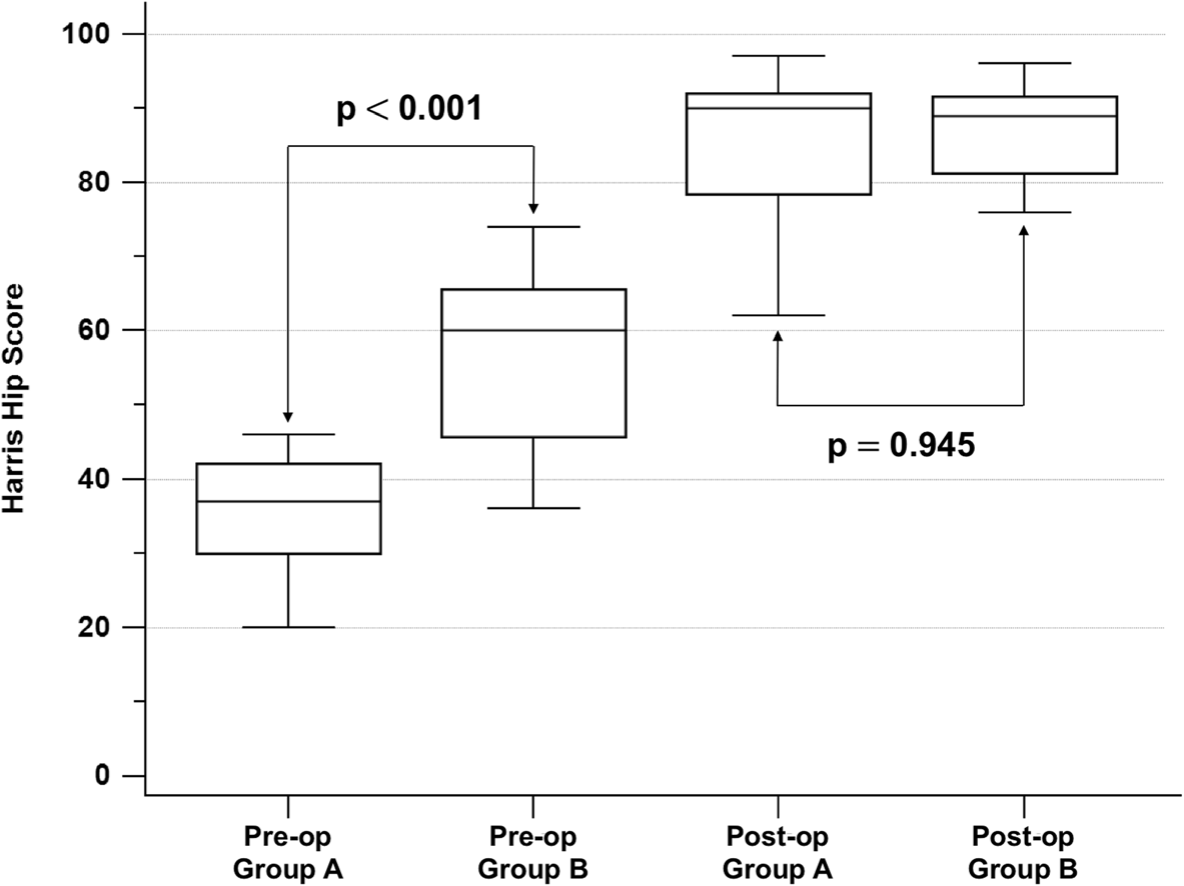
Box-and-whisker plots of preoperative and postoperative hip scores in the two groups

In the 29 patients who underwent reimplantation, the treatment success rate following reimplantation was 86.2%(25/29). The median period of follow-up was 29 months (IQR: 23–35.5). The patients in group A had a lower median HHS (37; IQR: 30–42 vs. 60; IQR: 45.75–65.5, *p*<0.001) before reimplantation than those in group B, while the two groups had a similar median HHS after reimplantation (90; IQR: 78.5–92 vs. 89; IQR: 81.25–91.5, *p*=0.945) at the latest follow-up (Fig. 5).

## Discussion

Various rates of complications associated with articulating PMMA spacers have been reported in recent literature (Table 4), but no study has provided valuable evidence to substantiate the risk factors for such complications [13, 18, 19]. To our knowledge, this study was the first to demonstrate the risk factors associated with development of mechanical complications of articulating spacers in two-stage revision hip arthroplasty. Patients who experience articulating spacer complications have decreased functional activity in the interim period and do not benefit from the heightened mobility that articulating spacers can provide. Although mechanical complications might require exchange of the articulating spacer in the interim period, most of our patients (78.6%, 11/14) did not undergo additional spacer exchange surgery, instead having a shortened interim period before reimplantation. Therefore, surgeons should aim to prevent the occurrence of mechanical complications of articulating spacers in order to maintain quality of life in the interim period for patients undergoing two-stage exchange arthroplasty.

**Table 4:** Literature regarding hip-spacer complications

Study	No. of patients	Spacer type	Spacer mechanical complications	Overall	Outcome after reimplantation
Duncan et al. (1993) [12]	13	PROSTALAC	3 dislocations	23%	1 allograft nonunion (8%) 1 loose Lord ring (8%) 2 heterotopic bones (15%)
Ivarsson et al. (1994) [26]	5	Hand-made	1 dislocation 1 subtrochanteric fracture	40%	–
Leunig et al. (1998) [18]	12	Hand-made	5 dislocations 1 spacer fracture	50%	–
Younger et al.(1998) [3]	29 (30 spacers)	PROSTALAC	3 dislocations	10%	1 extrusion of cement into knee (3%) 1 recurrent infection (3%) 2 recurrent dislocations (7%) 2 allograft nonunions (7%) 1 loose Lord ring (3%) 3 prosthesis migrations (10%)
Magnan et al. (2001) [27]	10	Mold	1 dislocation	10%	–
Koo et al. (2001) [28]	22	Mold	1 femur iatrogenic fracture	5%	1 recurrent infection (5%) 2 peroneal nerve palsies (9%) 1 greater trochanteric non-union (5%) 3 heterotopic ossifications (14%)
Durbhakula et al. (2004) [14]	20	Mold	2 dislocations 2 spacer fractures	20%	–
Hsieh et al. (2004) [4]	58	Mold	2 dislocations 2 spacer fractures	7%	2 recurrent infections (3%) 1 dislocation (2%)
Jung et al. (2009) [13]	82 (88 spacers)	Mold	15 spacer dislocations 9 spacer fractures 12 femoral fractures	40.9%	16 dislocations (23%)
Faschingbauer et al. (2015) [15]	138	93 mold 45 hand-made	12 dislocations 12 spacer fractures 1 femoral fracture 1 spacer fracture-dislocation 1 spacer protrusion into pelvis	19.5%	–
This study	35	Mold	6 dislocations 3 spacer fractures 1 spacer fracture-dislocation 4 femoral fractures	40%	3 recurrent infections 1 dislocation 1 greater trochanteric non-union

A primary finding of our study was that younger patients were much more likely to suffer mechanical complications than older patients. The high demand placed on the spacer during daily activity in younger patients may significantly predispose them to excess stress, leading to mechanical complications (i.e., spacer fractures). In our study, the metallic endoskeletons were composed of three large Kirschner wires. Such types of metallic endoskeleton may predispose spacers to fractures [18]. Hsieh et al. reported two fractures of the cement femoral stem in 24 patients (8.3%), which was similar to our study (9.7%) [20]. Given that younger patients have higher functional demands in daily activity, stronger metallic endoskeletons, such as preformed spacers with reinforced metal cores [12, 21], should be considered to provide mechanical integrity and minimize the risk of spacer fracture.

Chronic PJI was also identified as a primary risk factor for mechanical complications in the patients with articulating spacers. Individuals with chronic infections locally are subject to deleterious bone destruction from chronic inflammation, which may propagate defects detrimental to spacer fixation at the joint [3, 20, 22]. As there was no significant difference in Paprosky classification between groups A and B in our study, we believe that chronic PJI of the hip may result in poor bone quality, leading to mechanical complications of articulating spacers. Additionally, although all study patients underwent the same standard rehabilitation program following spacer implantation, iatrogenic femur fractures only occurred in the interim period in patients with chronic PJI.

In our study, we observed spacer-related complications in the majority (92.9%) of patients who underwent the procedure via the posterior approach. Recent literature has described the posterior approach as resulting in a higher dislocation rate in comparison to the direct lateral approach in THA [23, 24]; however, with meticulous and secure closure of the posterior capsule, the dislocation rate can be lowered to a rate comparable to that seen in patients in which the direct lateral approach is used [25]. Patients with PJI require extensive debridement, and this may present a problem with regards to adequate closure of the posterior capsule, which may lead to instability. Therefore, we hypothesize that the dislocation rate following spacer implantation after PJI may increase with use of the posterior approach.

The success rate for infection eradication under the two-stage revision protocol is around 82 to 96% [3–8], and our result (86.2%) was consistent with previous literature. Furthermore, the clinical outcome was not different in the two study groups following reimplantation.

There were several limitations to our study that we would like to acknowledge. The first was regarding the retrospective nature of the study, in addition to the small study cohort. The cohort size resulted from our institution’s patient cohort treated with articulating spacer implantation. Second, we did not stratify each category of spacer complication owing to the small cohort size. Finally, we were unable to measure the precise amount of time following which each patient could bear weight on the implant, which may have contributed to the occurrence of spacer-related complications.

## Conclusion

Our study demonstrated a high complication rate for articulating PMMA spacers in two-stage revision hip arthroplasty. We identified a young age and chronic PJI as the primary risk factors for development of mechanical spacer complications. Surgeons should remain cautious when using articulating spacers in patients with cranial defects of the acetabulum and those who have reduced containment of the spacer within the hip joint. Further research is needed in order to eliminate spacer-associated complications in order to provide a better patient experience during the interim stage.

## Data Availability

Data are available from the corresponding author.

## Abbreviations

ASA: American Society of Anesthesiologists
BMI: Body mass index
CI: Confidence interval
CRP: C-reactive protein
ESR: Erythrocyte sedimentation rate
Hb: Hemoglobin
HHS: Harris hip score
IQR: Interquartile range
OD: Odds ratio
PJI: Periprosthetic joint infection
PMMA: Polymethylmethacrylate;
SD: Standard deviation
THA: Total hip arthroplasty
